# Is prostate cancer radiotherapy using implantable rectum spacers safe and effective in inflammatory bowel disease patients?

**DOI:** 10.1016/j.ctro.2021.01.007

**Published:** 2021-01-25

**Authors:** Ben G.L. Vanneste, Evert J. Van Limbergen, Tom Marcelissen, Kobe Reynders, Jarno Melenhorst, Joep G.H. van Roermund, Ludy Lutgens

**Affiliations:** a Department of Radiation Oncology (MAASTRO), GROW – School for Oncology and Developmental Biology, Maastricht University Medical Center+, Maastricht, the Netherlands; b Department of Urology, Maastricht University Medical Center+, Maastricht, the Netherlands; c Department of Surgery, Maastricht University Medical Center+, Maastricht, the Netherlands

**Keywords:** Prostate cancer, Radiotherapy, Rectum balloon implant, Inflammatory bowel disease

## Abstract

**Background:**

Prostate cancer radiotherapy (RT) in patients with (active) inflammatory bowel disease (IBD) remains controversial. We hypothesized that RT in combination with a biodegradable prostate-rectum spacer balloon implantation, might be a safe treatment approach with acceptable toxicities for these high risk for rectal toxicity patients.

**Materials and methods:**

We report on a small prospective mono-centric series of 8 patients with all-risk prostate cancer with the comorbidity of an IBD. Four patients had Crohn’s disease and 4 patients had ulcerative colitis. One out of four had an active status of IBD. All patients were intended to be treated with curative high-dose RT: 5 patients were treated with external beam RT (70 Gray (Gy) in 28 fractions), and 3 patients were treated with ^125^I-implant (145 Gy). Toxicities were scored according to the CTCAE v4.03: acute side effects occur up to 3 months after RT, and late side effects start after 3 months.

**Results:**

Median follow-up was 13 months (range: 3–42 months). Only one acute grade 2 gastro-intestinal (GI) toxicity was observed: an increased diarrhea (4–6 above baseline) during RT, which resolved completely 6 weeks after treatment. No late grade 3 or more GI toxicity was reported, and no acute and late grade ≥2 genitourinary toxicity events were observed.

**Conclusion:**

Prostate cancer patients with IBD are a challenge to treat with RT. Our results suggest that RT in combination with a balloon implant in selective patients with (active) IBD may be promising, however additional validation is needed.

## Highlights

• Radiotherapy in patients with inflammatory bowel disease remains controversial.
• A biodegradable balloon is inserted between the prostate and the rectal wall.
• The balloon pushes the anterior rectal wall outside of the high-dose area.
• No grade 3 or more rectal toxicities were observed.

## 1 Introduction

Prostate cancer is the most common cancer among males in the Western world with a lifetime risk of 1 in 9 [1]. Localized prostate cancer is currently treated by a radical prostatectomy or high-dose external beam radiotherapy (EBRT) and/or brachytherapy with equal outcomes but different toxicity profiles [2].

Patients with inflammatory bowel disease (IBD), i.e. ulcerative colitis or Crohn disease, are considered to have a high risk for rectal complications due to pre-existing inflammation, fibrosis, adhesions and scarring in the pelvic region which exacerbate the EBRT-linked toxicities. In the literature, no clear consensus exists on what is the best treatment approach for these patients as results are conflicting [3], [4], [5]. Tolia et al. reported on 3 publications with an acute grade 3 or more GI complications attributable to RT to 21% of the treated patients. A late grade 3 or more GI toxicity was developed in a range between 8 and 29%. Tromp et al reported on 8 publications with a reported acute grade 3 or more GI toxicity in 20% of patients receiving EBRT and in 7% of patients receiving brachytherapy. Late grade 3 or more GI toxicity occurred in 15% of EBRT patients and in 5% of patients receiving brachytherapy. These high reported toxicities are higher than in regular patients, however, it is not unambiguous that IBD corresponds to high toxicity rates, but there is an increased risk to develop these toxicities. Some groups described even very high toxicity rates: an increase in Grade ≥3 gastro-intestinal (GI) toxicity up to 73% [3], or increased risk of adhesions and surgical fistulae [6]. Because of multiple abdominal surgeries typically occurring in these patients, many prostate surgeons are reluctant to propose prostatectomy for these patients. As a result many patients are left eventually between Scylla and Charybdis: or they choose to face (a high risk of) dreadful toxicities, or they accept only palliative treatment for their cancer.

Several medical devices have been developed to decrease the RT dose on ano-rectal structures to decrease the GI complication risk [7]. Implantable rectum spacers (IRS) push the rectum out the high-dose RT region by injection of an absorbable hydrogel, hyaluronic acid, a collagen implant, or a saline-filled rectal balloon implant (RBI) [8]. These studies have demonstrated that an IRS effectively decreases the anorectal dose with subsequently decreasing acute and late rectal toxicity and increasing the cost-effectiveness [9], [10]. When patients can effectively be selected upfront at high-risk of rectal toxicities, cost-effectiveness of IRS will be further improved [11], [12]. This series is a specified selection of patients with IBD who have *a priori* a very high risk of rectal toxicities resulting RT. The aim of this prospective report was to investigate early and late toxicities in a small series of patients with IBD, receiving RT (EBRT or Brachytherapy) in combination with RBI to avoid high radiation dose to the overlying bowel.

## 2 Patients and methods

### 2.1 Patient selection

Patients with a histologically confirmed, loco-regionalized (stage cT1-3N0-1) adenocarcinoma of the prostate and with a histologically proven IBD status were prospectively enrolled in this report between 2016 and 2019 to receive an RBI (BioProtect Ltd, Israel) with a RT treatment. The patient and tumor characteristics are summarized in Table 1. The flare-up of IBD status was no exclusion criteria; 25% was in relatively active status. Four patients determined as intermediate-risk were prescribed additional neo-adjuvant androgen suppression therapy for 6 months. The high-risk patients were offered an additional 1.5 years androgen suppression therapy in extent of the 6 months neo-adjuvant therapy before EBRT. All patients underwent magnetic resonance imaging (MRI) to exclude dorsal extra-prostatic extension (stage dorsal T3a/4), which was an exclusion criterion. Regional extension was not an exclusion criterion. A Prostate Specific Membrane Antigen-Positron Emission Tomography (PSMA-PET) was performed in the high-risk patients to exclude distant metastasis. This study was performed under institutional review board approval.

**Table 1:** Patient (N = 8), IBD and tumor characteristics.

*Age* (years; median [range])	72 [69–76]
Prognostic risk group*: (no. of patients)	
1 Low-risk	1 (13%)
2 Intermediate-risk	4 (50%)
3 High-risk	3 (37%)
**IBD:**	
*Cröhn*	4 (50%)
*Colitis*	4 (50%)
**Involving rectum**	6 (75%)
**IBD activity:**	
*Quite*	6 (75%)
*Active*	2 (25%)
**Previous abdominal/pelvic surgery**	5 (63%)
**Treatment:**	
*External Beam Radiotherapy*	5 (63%)
*Brachytherapy LDR*	3 (37%)

*Abbreviation: IBD:* Inflammatory Bowel Disease.

*Low-risk: no risk factors: PSA < 10 ng/ml; Gleason score <7; cT-stage <2b; *Intermediate-risk:* PSA 10–20 ng/ml and/or Gleason score = 7 or cT-stage = 2b/c; *High-risk:* PSA > 20 ng/ml or Gleason score >7 or cT-stage >2b/c.

### 2.2 RBI implantation procedure

In case of external beam RT, 4 fiducial markers (PolyMark™, CIVCO, Orange City, USA) were implanted intra-prostatically for image guidance during the treatment. Afterwards, a RBI was implanted between the prostate and the anterior rectal wall 10–12 days prior to the start of the EBRT treatment.

In case of brachytherapy, the RBI was inserted after the seed implantation to avoid RBI puncturing by brachytherapy needles. All RBI and brachytherapy procedures were performed by the same radiation oncologist (BV). The RBI implantation technique has already been described in detail previously [13]. The RBI was implanted transperineally under bi-plane transrectal ultrasonography guidance and was inflated by a bubble-free (sterile) saline solution.

### 2.3 Target volume definition, dose prescription and treatment planning

Each patient underwent a CT scan and MRI scan 5–7 days after RBI implantation in supine position with a slice thickness of 3 mm. A bladder protocol was advised daily during external RT and before image acquisition: patients were instructed to empty their bladder, then they were asked to drink 300 ml of water to have a filled bladder. Laxatives were not recommended. The CT and MRI scans were co-registered based on the fiducial markers.

**Fig. 1:**
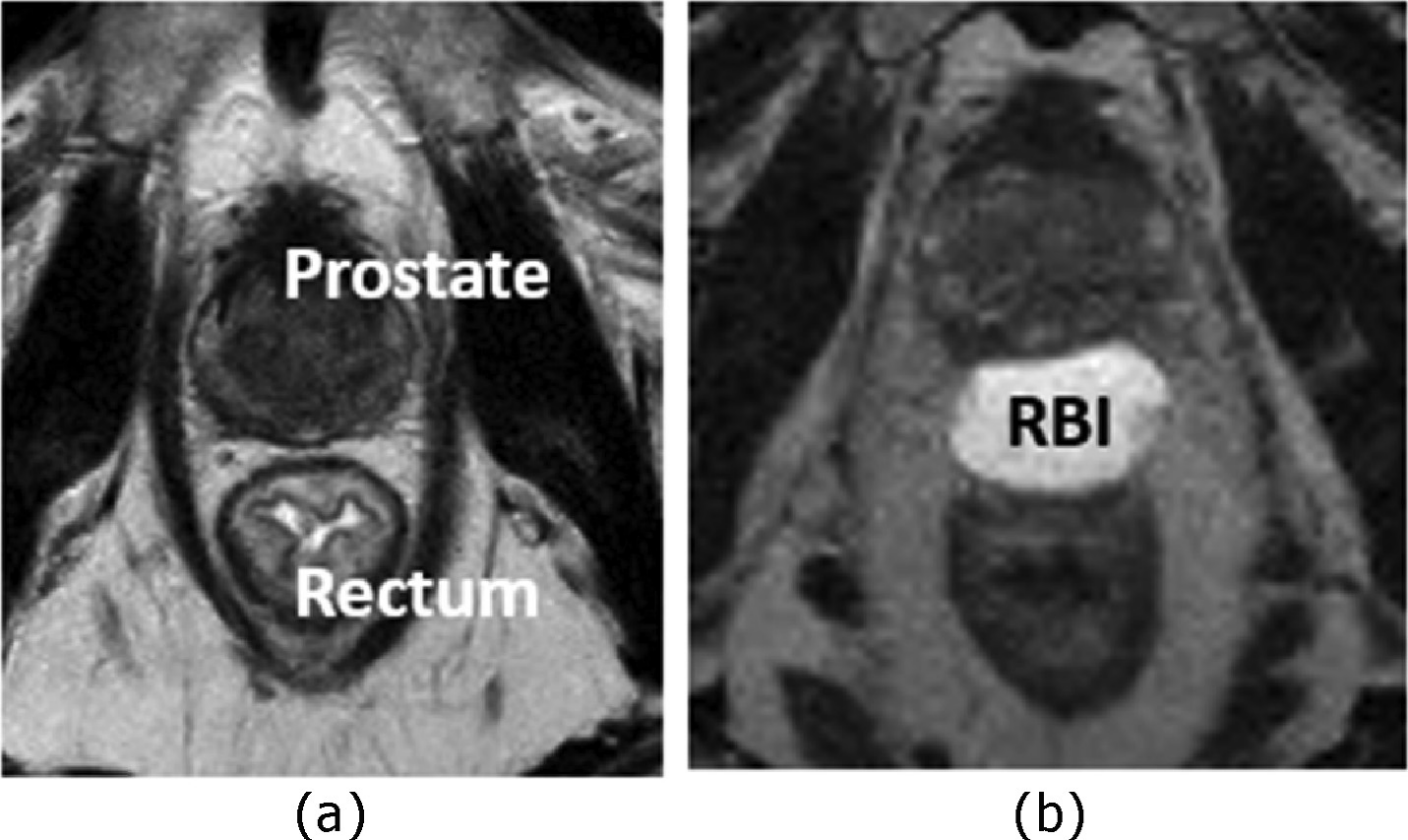
Axial T2-weighted MRI of a patient with a RBI before (a) and after implantation (b). *Abbreviation*: MRI = Magnetic Resonance Image; RBI = Rectal Balloon Implant.

**Fig. 2:**
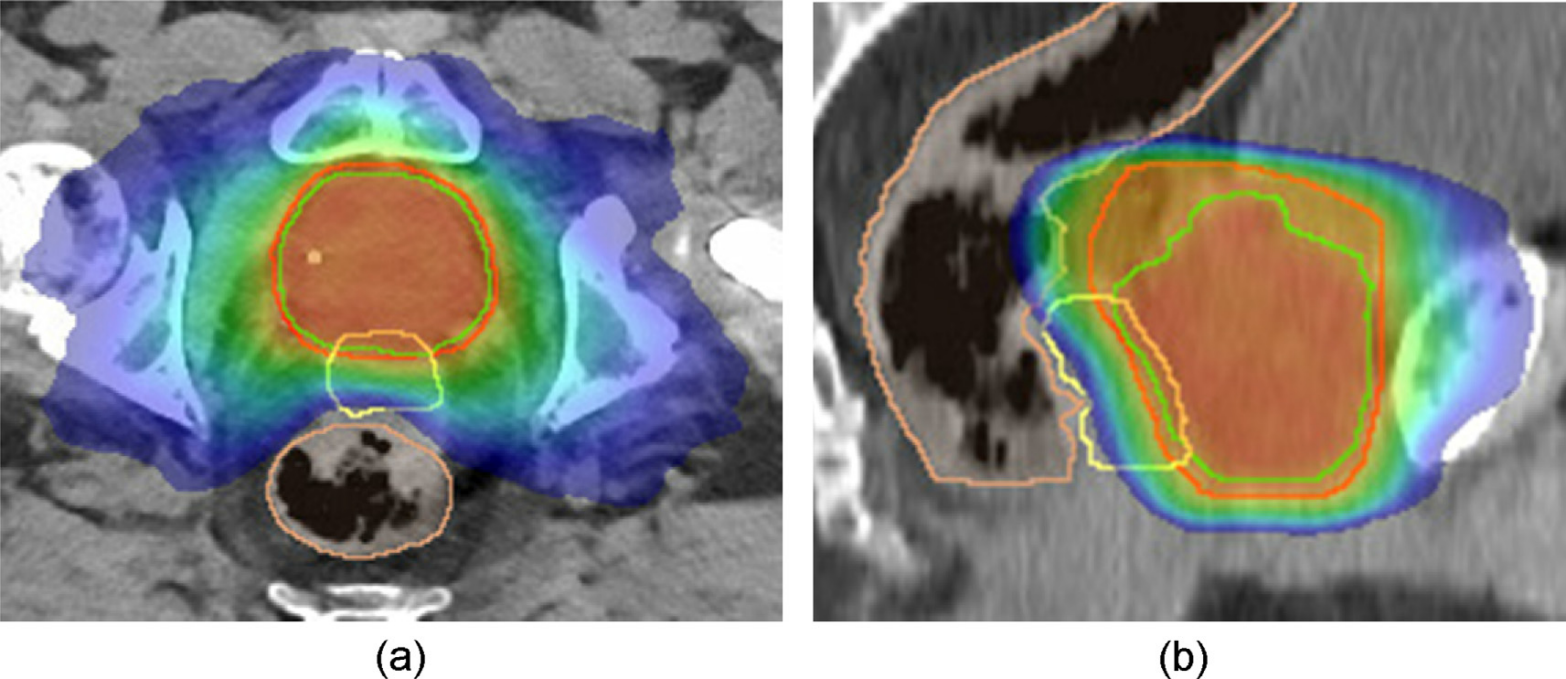
Isodose distribution in an axial (a) and sagittal (b) CT plane in color-wash is presented after RBI (yellow) implantation, with prostate PTV1 (red), PTV2 (green). The prescribed dose to PTV1 and PTV2 was 65.8 and 70 Gy, in 28 fractions of 2.35 and 2.5 Gy, respectively. The low- and intermediate-dose region >35% (blue) with nearly no overlap in the rectum (brown) is illustrated in image (a). The high-dose region >80% (green) reveals no overlap at all within the rectum. In image (b) at the more cranial part there is minimal overlap observed of the low-intermediate-dose region. *Abbreviation*: RBI = Rectal Balloon Implant; PTV = Planning Target Volume. (For interpretation of the references to color in this figure legend, the reader is referred to the web version of this article.)

The T_2_-weighted MRI scan were used for delineation of the prostate and seminal vesicle (CTV = clinical target volume), while the RBI and the organs at risk were delineated on the CT scan (Fig. 1). The first planning target volume (PTV1) was constructed according to the institutional protocol by expanding the CTV with 10, 7 and 6 mm in cranial-caudal, anterior-posterior, and left–right direction respectively. A second PTV (PTV2) was defined as a 5 mm isotropic expansion of the CTV, with exclusion of the bladder, anal canal and rectum. A volumetric modulated arc technique (VMAT) using two half gantry rotations of 10 MV photon beams are planned for dose delivery (Eclipse Version ICD-10, Varian Medical Systems Inc., Palo Alto, USA) (Fig. 2). The prescribed dose to the planning target volume (PTV) was 70 Gy, in 28 fractions of 2.5 Gy. One patient with a regional lymph node metastasis, received pelvine radiotherapy in 28 fractions of 1.8 Gy. With α/β = 3 Gy for late rectal toxicity, the maximum 2 Gy equieffective dose (EQD_2_(3)) in the anorectum for this type of plan is 77 [14]. The overall treatment time was 7 weeks, at 4 fractions a week. The dose-volume constraints fulfilled the institutional protocol, which is based on the QUANTEC guidelines [15]. All patients underwent daily X-ray based position verification and repositioning based on the intraprostatic fiducial markers.

**Fig. 3:**
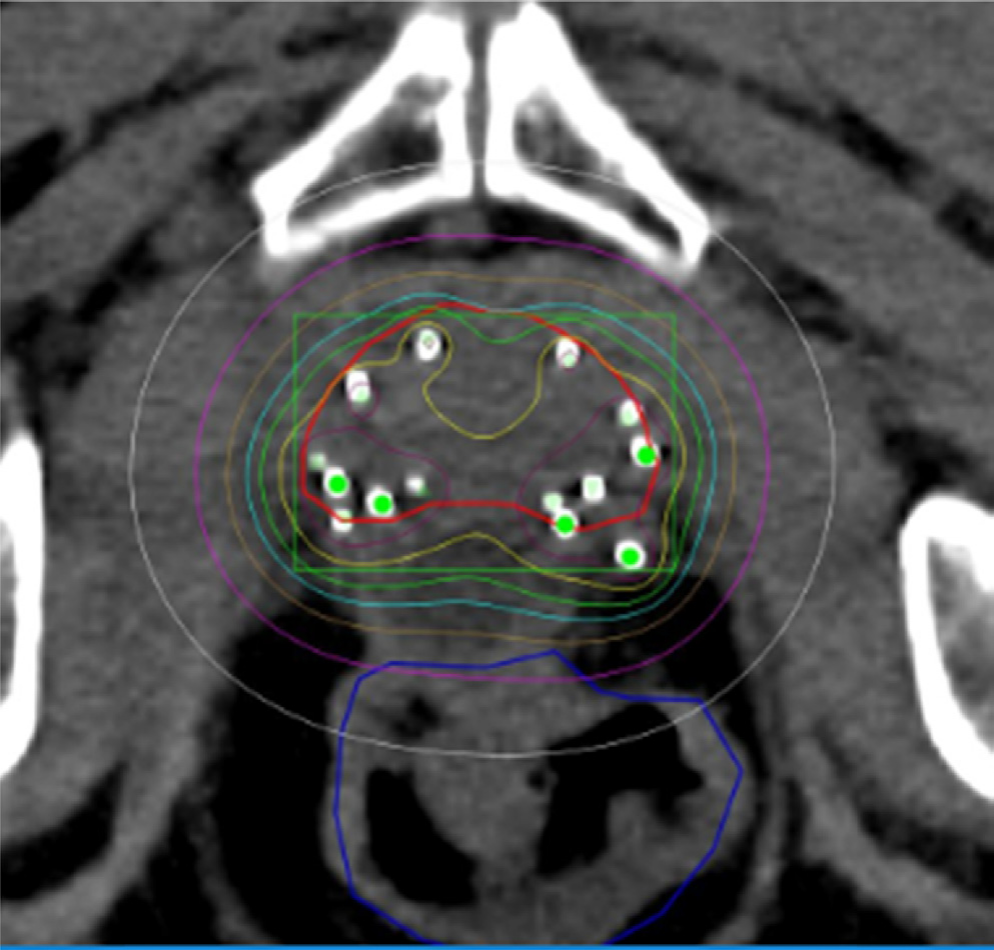
Isodose distribution in an axial CT plane after ^125^I brachytherapy implantation with a RBI. The prescribed dose to the prostate (red) was 145 Gy (green). The high-dose region >120% (yellow) and the low-dose region <40% (purple) with minimal overlap in the rectum (dark-blue). *Abbreviation*: RBI = Rectal Balloon Implant. (For interpretation of the references to color in this figure legend, the reader is referred to the web version of this article.)

The brachytherapy was performed with ^125^I seeds and the prescribed dose to the prostate gland was 145 Gy (Fig. 3).

Weekly imaging by Cone Beam CT scans during the course of EBRT treatment is performed to assess possible deflation dynamics of the RBI, and to confirm the prostate–rectum distance sustained more than 1 cm. For the brachytherapy, a dosimetry CT scan is performed one month after implant, with also a check of the integrity of the RBI.

### 2.4 Toxicity

Complications were assessed in terms of Common Terminology Criteria for Adverse Events (CTCAE) (version 4.03): acute toxicity criteria for GI, genitourinary (GU), and erectile dysfunctions [16]. Acute side effects occur up to 3 months after RT by definition. Chronic side effects start after 3 months, up to 6 months or even years later after RT. The first follow-up appointments were 4 weeks after treatment. Patients were observed every 3–6 months for 2 years, and then 6 months or annually thereafter.

## 3 Results

The median follow-up was 13 months [range: 3–47 months]. The median injected RBI volumes were 12.0 cm^3^ [range: 10–17 cm^3^]. All patients are alive with no evidence of biochemical or clinical recurrence of prostate cancer. Median PSA at last follow-up was 0.2 ng/ml [range: 0–0.4].

One patient was intended to treat, however no treatment was delivered. The seed implantation failed and was aborted because of pubic arc interference. Moreover, during the preparation of the brachytherapy seeds implantation the ultrasound probe was iatrogenic installed into the anastomosis of the ileo-anal pouch with a partial rupture as result. The pouch was complete repaired by a colorectal surgeon (JM) without sequelae.

In the irradiated patients, only one grade 2 GI toxicity was observed: an increased diarrhea-level (>4–6 times above baseline) was reported during RT. However, patient remained ambulatory, and a complete remission of the complaints was observed at 6 weeks after treatment. Two patients got a colonoscopy in the context of a suspicion of flare-up of the IBD status. In both patients no tissue damages of the distal parts of the rectum were observed, and in one patient the flare-up was confirmed. This patient had a ulcerative colitis flare-up 6 months after RT, which was also confirmed with increased calprotectin levels in the faeces. Calprotectin is a protein found in neutrophil granulocytes, and is an indication for an inflammatory diarrhea. After flare-up, condition recovers well. Three patients (45%) had no GI toxicity at all.

One patient reported grade 1 GU toxicity, consisting of dysuria, pollakisuria, and nycturia. Six patients (85%) had no GU toxicity. Urinary retention was not observed.

## 4 Discussion

IBD is characterized by chronic inflammation of the GI tract. It is considered as a relative contraindication to abdominal radiotherapy because of the increased toxicities observed, whereas an active inflammatory episode of IBD is considered as an absolute contraindication. Avoiding radiotherapy associated toxicity by avoiding dose to the involved organ is a rational strategy [3], [4], [5]. A good option in these patients would be to consider therapeutic strategies such as radical prostatectomy, avoiding any radiation exposure of bowel at all. However, often surgery is not a valuable option, because many patients already underwent multiple abdominal procedures. Moreover, certainly in case when radical surgery seems not feasible, postoperative radiotherapy of the prostate bed becomes indicated at least following biochemical progression. Although active surveillance is feasible in many good risk prostate cancer patients, this strategy is in general not an acceptable option for younger patients with an increased risk prostate cancer. Furthermore, many patients initially followed within an active surveillance protocol, eventually need treatment because of disease progression. Therefore many of these patients eventually will have radiotherapy as the single treatment option.

A few papers reported on the combination of RT and IBD patients:

Conventional EBRT techniques describe mean Grade ≥3 GI toxicities ranging from 15% to 73% [3], [4], [5]. These authors concluded that the location and the activity status of the IBD in combination with the EBRT bowel dose and volume (which is related to the technique) are determining the severity of post-irradiation toxicity. Therefore some advocate the use of Brachytherapy as a means to diminish the dose to the rectum. However, for LDR (low-dose-rate) brachytherapy (I^125^), the mean rate of acute and late grade 3 or more GI toxicity remains significant 23% and 15%, respectively [17]. Two patients (15%) developed peri-anal fistulas and needed surgical repair. Both patients had active ulcerative colitis at time of treatment. They concluded that LDR brachytherapy should be used with great caution or even avoided for men with active IBD involving the rectum.

Alternatively, for HDR (high-dose-rate) brachytherapy, the toxicity profile seemed more favorable, [18]. Grade 1 proctitis was reported in 27%, grade 1 diarrhea in 9%. No ≥grade 2 GI toxicity was reported [18]. They concluded that HDR could be a possible attractive approach for IBD prostate cancer patients. They speculated that the differences in observed toxicity between HDR and LDR brachytherapy could be explained by inherent differences between both techniques: real-time imaging and treatment delivery in case of HDR, which is more reliable and convenient for rectal dose constraints than LDR brachytherapy where dose is delivered over many months, allowing shifts in the position of the seeds, and deformations of the prostate to alter dosimetry [19]. However, these techniques are not randomized examined, and it is too speculative to demonstrate this. This should be further examined in a randomized research.

In our series, we propose another workaround to increase safety RT treatment in these patients by pushing the ‘unhealthy/inflammatory’-predisposed rectum out the high-dose region by a RBI. On the basis of our small series this approach seems safe and effective.

It is reasonable to assume that IBD comorbidity might increase the risk of complications of RBI placement. Theoretically one might expect that dissection of the plane between the prostate and the rectum might become impossible due to post-inflammatory fibrosis and scarring. We did not observe this problem in our small series of patients. Alternatively increased risk of perforation of the rectal wall might be present, due to deep ulcerations, inflammatory processes or postoperative changes compromising the integrity of the rectal wall. We did observe a tear of an ileo-anal pouch caused by excessive pressure of the ultrasound probe in a blind ending loop. This toxic risk applies to any form of transrectal ultrasound procedure, including prostate biopsies, fiducial placement, LDR seeds of HDR brachytherapy, and also for RBI placement. To our knowledge no data of prevalence is known. In case of ileo-anal pouches we advise to perform transrectal probes with extreme caution using, and only in experienced hands. Moreover a previous evaluation with colonoscopy with ultrasound can be performed in advance to identify as many problems as possible. Such interventions should be preferentially not performed under general anesthesia.

Additional possible disadvantages of a RBI implantation such as infection, thrombosis, or dysuria are not observed in these small series.

Additional testing for IBD status can be important element in the workup of these patients. Calprotectin can be measured in the faeces, and correlates to the activity of inflammation. In this context, Calprotectin is used to monitor therapeutic responses in IBD patients to anti-inflammatory drugs [20]. Calprotectin and lactoferrin faecal values changing during RT treatment and after cessation of RT, with correlation to acute and chronic proctitis symptoms in 75% of the patients [21]. As the grade of IBD-activity is predictive for radiation induced toxicity, it might be a wise approach to postpone radiation treatment until IBD activity is lowered at a later time, although good evidence is lacking. In intermediate and high risk prostate cancer patients, hormonal therapy might be used to bridge this gap.

Furthermore, it is important to take into account that differences in rectal filling can be observed by inclusion of gas bubbles and/or stool. These situations can influence the distance between the prostate and the rectum: at the lower level of the rectum the distance is still enlarged by the presence of the RBI, however at the higher level (the cranial part) the distance could decrease incrementally. This is especially the case when the rectal filling is increased dramatically, and when seminal vesicles are irradiated. This means theoretically that the possible advantageous aspects on the dose distribution with a RBI can be nullified by still a significant dose more cranially, especially when larger volumes are irradiated.

This study has several limitations. Firstly, the follow-up period (median 13 months) is too short for drawing definite conclusions on long term toxicity safety profile. Secondly, our series consist of a limited group of patients. Thirdly, to report RT toxicities and compare these with other, older studies is not unambiguously: the previously reported toxicities can also be the result of an active status or flare-up of IBD, even without RT. Finally, most literature of the spacers is limited to patients with low-and intermediate-risk prostate cancer. The role of spacers in locally advanced and high-risk prostate cancers regarding potential rectal wall invasion is not yet clear. Villers and colleagues described prostate cancer invasion in the Denonvilliers’ fascia of 19% of cases in their series of 243 prostatectomy specimens [22]. No tumor invades completely through the full thickness of this structure. The possible negative influence of a spacer in cases with a dorsal prostate capsule rupture (cT3a) is unclear, as tumor cells could be displaced out of the high-dose region by the spacer. More research is needed to determine the role of spacers in these patients.

Our series illustrate a possible treatment strategy for patients with IBD in need of curative prostate cancer RT. This treatment approach of RT in combination with an IRS could in our opinion be considered in carefully selected patients as a treatment option in this specified high-risk on toxicities patient population to obtain good outcome with acceptable toxicity results. Further research is mandatory to evaluate the complete role of spacers in these group of patients.

## 5 Conclusion

Our results suggest that RT in patients with IBD in combination with biodegradable RBI may be safe, effective and promising, however more data is required to confirm this result.

## Patients’ rights

All procedures followed were in accordance with the ethical standards of the responsible committee on human experimentation (institutional and national) and with the Helsinki Declaration of 1975 (in its most recently amended version). Informed consent was obtained from all patients included in the study.

## Ethical statements

As the corresponding author, I declare that the work described in the manuscript is unpublished, and is not concurrently being considered for publication elsewhere. I can confirm that the manuscript has been read and approved by all named authors and that there are no other persons who satisfied the criteria for authorship but are not listed. I further confirm that the order of authors listed in the manuscript has been approved by all of us.

## Declaration of Competing Interest

The authors declare that they have no known competing financial interests or personal relationships that could have appeared to influence the work reported in this paper.
